# Reference frame selection in dialog: priming or preference?

**DOI:** 10.3389/fnhum.2013.00667

**Published:** 2013-10-16

**Authors:** Katrin Johannsen, Jan P. De Ruiter

**Affiliations:** Faculty of Linguistics and Literary Studies, Bielefeld UniversityBielefeld, Germany

**Keywords:** spatial perspective, priming, spatial frames of reference, psycholinguistic, dialog

## Abstract

We investigate effects of priming and preference on frame of reference (FOR) selection in dialog. In a first study, we determine FOR preferences for specific object configurations to establish a baseline. In a second study, we focus on the selection of the relative or the intrinsic FOR in dialog using the same stimuli and addressing the questions whether (a) interlocutors prime each other to use the same FOR consistently or (b) the preference for the intrinsic FOR predominates priming effects. Our results show effects of priming (more use of the relative FOR) and a decreased preference for the intrinsic FOR. However, as FOR selection did not have an effect on target trial accuracy, neither effect alone represents the key to successful communication in this domain. Rather, we found that successful communication depended on the adaptation of strategies between interlocutors: the more the interlocutors adapted to each other's strategies, the more successful they were.

## INTRODUCTION

**FIGURE 1:**
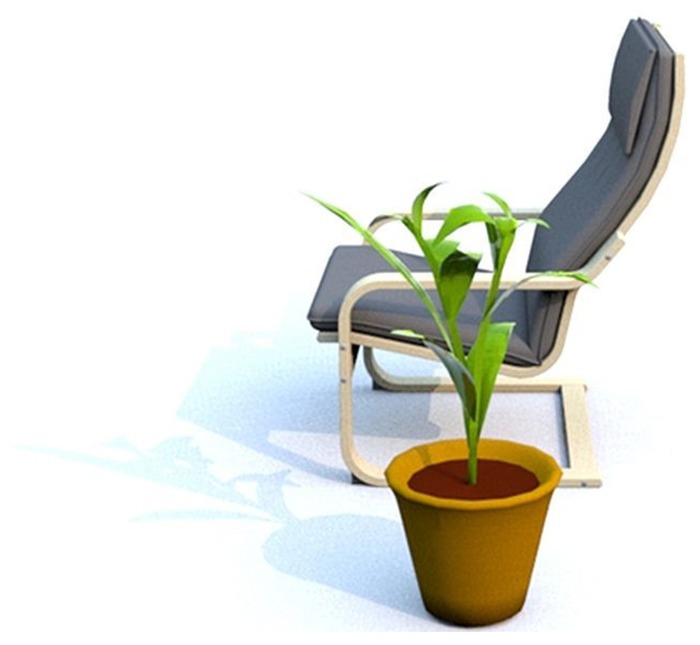
**Reference object (armchair) rotated 90° with located object “in front of it” (relative reference frame) or “to the left of it” (intrinsic reference frame)**.

Localizing an object with reference to another object is common in natural language. For instance, consider the sentence “The book is to the left of the chair.” It is ambiguous whether the book is at the chair’s left or whether it is to the left of the chair as viewed from the speaker’s perspective. In order to refer to these different perspectives, frames of reference (FOR) are used. FOR are a set of axes that parse space (Carlson, 1999) and can be considered as coordinate systems that impose an orientation on the environment, people, or objects. These coordinate systems have an origin constituted by the point of intersection (Miller and Johnson-Laird, 1976), a direction and an orientation (Logan and Sadler, 1996). Following Levinson (1996, 2003), three different FORs can be distinguished which differ with regard to their origin and the spatial relationship they establish. The *relative *FOR establishes viewpoint-dependent ternary spatial relationships. A description of the object configuration in **Figure 1** according to the relative FOR would be “The plant is in front of the chair”; the spatial relationship comprises the plant, the chair and the viewer. In the present study, the origin of the relative FOR always lies in the viewer; the coordinate system is thus oriented egocentrically and the spatial relationship comprises the speaker’s viewpoint and two objects. In the case of the *intrinsic* FOR, the relationships are binary and viewpoint-independent. The intrinsic FOR is used when the origin lies in the object itself and the direction of the FOR is oriented according to the inherent axes of the object (“The plant is next to the chair” in **Figure 1**). The *absolute* FOR is based on environmental features such as gravity or the cardinal directions and will not be considered in this study.

Situations in which the relative and the intrinsic FOR can be used interchangeably exhibit a high potential for ambiguities. If both FOR are equally likely to be used and speakers do not indicate which FOR they are using, the probability that an interlocutor interprets the FOR correctly is at chance level. Attempts to define preferences for specific FORs have led to ambiguous results. The relative FOR, being perceptually available and avoiding the extra computational effort needed for mental rotation, has been considered predominant by some authors (Linde and Labov, 1975; Levelt, 1982, 1989) whereas other authors have claimed that the intrinsic FOR predominates (Miller and Johnson-Laird, 1976) or is at least preferred (Carlson-Radvansky and Irwin, 1993; Carlson-Radvansky and Radvansky, 1996; Taylor et al., 1999). This disagreement and the potential for ambiguities has led to an extensive body of psycholinguistic investigations of which factors contribute to the selection and processing of spatial FOR, mostly using monolog studies. The factors identified range from functional relations between objects (Carlson-Radvansky and Radvansky, 1996) to motion characteristics (Levelt, 1984), gravity (Friederici and Levelt, 1990), priming effects (Watson et al., 2004; Carlson and Van Deman, 2008; Johannsen and de Ruiter, 2013), scene type (Johannsen and de Ruiter, 2013), and properties of the object configuration such as the rotation of the reference object and the position of the located object (Ziegler et al., 2012).

However, monolog studies do not allow us to investigate how interlocutors deal with FOR ambiguities in dialog. Dialog differs from monolog in that dialog “language use is really a form of joint action” (Clark, 1996, p. 3) which suggests that FOR interpretation in dialog requires that interlocutors coordinate FOR selection in order to communicate successfully. Even though there have been attempts to investigate spatial perspective taking involving an imagined interlocutor (Herrmann, 1988; Duran et al., 2011), studies using real dialogs are rare. Perspective taking between interlocutors who have different physical vantage points and thus different perspectives on the same scene is not considered in the present study, but interesting results can be found elsewhere (e.g., Bürkle et al., 1986; Schober, 1993; Galati et al., 2013). Watson et al. (2004) showed that dialog partners tended to align on FORs, revealing a tendency to use the same FOR that their interlocutor had previously used. This was interpreted as a case of alignment resulting from priming effects. Pickering and Garrod (2004, p. 173) claim that alignment is a key factor for successful communication and results from priming which is “essentially resource-free and automatic.” However, Watson et al. (2004) used a confederate as one of their participants, and the confederate’s utterances were scripted. Assuming that people do not merely adopt the interlocutor’s strategies but rather mutually influence one another in dialog, a confederate may not represent a natural dialog counterpart. Thus, FOR selection in a real dialog with two naïve participants may reveal different effects.

However, following the attempts to specify FOR preferences (as described above), it has been shown that there is a general preference for the intrinsic FOR in specific object configurations (Ziegler et al., 2012). This study also demonstrated an effect of the located object’s position with regard to the reference object’s FOR. If the located object was positioned on the front/back axis of a FOR, this made the selection of the respective FOR more probable. Thus, these axis-dependent preferences may reduce variability in FOR selection independent from priming effects. Furthermore, the general preference for the intrinsic FOR may lead interlocutors to establish a conceptual pact (comparable to conceptual pacts in lexical choices as discussed by Brennan and Clark, 1996) and use it consistently. However, in such cases, conflicts may arise from the opposing impacts of priming and preference of FOR.

The interaction between preference and priming effects has not, to our knowledge, previously been investigated. We expect that if automatic priming is a prevailing effect in conversation that leads to FOR alignment (cf. Pickering and Garrod, 2004; Watson et al., 2004), this should override FOR preferences. However, if FOR preferences lead to conceptual pacts with regard to FOR selection, this may override priming effects.

To investigate effects of preference and priming in dialog, we developed a priming study in which pairs of naïve participants described pictures of object configurations to each other. In each round, one of the participants was the director, who described a spatial configuration displayed on a monitor while the other participant (matcher) had to choose between two displayed pictures. While half of the stimuli only allowed the use of the relative FOR (prime trials), the other half consisted of stimuli allowing the use of the intrinsic FOR (target trials). After every two trials, the roles changed and the matcher became director. Thus, hearing the interlocutor A use the relative FOR in the prime trial should, according to the priming account, prime interlocutor B to select the relative FOR in the target trial.

## STUDY I: FOR PREFERENCES

In order to be able to separate preference from priming effects, we conducted a study in which we determined FOR preferences for specific object configurations as a baseline for comparisons.

### MATERIALS AND METHODS

As described above, FOR preferences are highly dependent on the context and object features. For this reason, we focused on objects from a single category (furniture). Spatial verbal descriptions were elicited in an online experiment in which participants were shown pictures of object configurations and were instructed to define the spatial relations by inserting spatial terms in gapped sentences. Their FOR preferences served as a baseline in the dialog study.

#### Participants

244 participants were recruited by email invitation. Data from 34 participants had to be excluded (due to a cease of participation or different native language), thus, data from 210 participants (168 women, 42 men) with a mean age of 24.1 years (ranging in age from 7 to 72 years) were used for analysis.

#### Stimuli and design

Stimuli were pictures of object configurations and German gapped sentences of the form “<located Object> steht —<reference object>.”**(“<located Object> stands —<reference object>.”). Thus, participants had to insert a spatial preposition and an article to fill the gap. Pictures were created using Sweet Home 3D, an architectural design software. 66 pictures were created, each consisting of a reference object and a located object. Different orientations of the reference object resulted from rotating it clockwise at angles of 90°, starting at 0° (reference object faces the observer). The located object (a plant or a stool) was placed in four different positions: relatively in front of, to the left of, behind and to the right of the reference object. This led to potential ambiguities in the descriptions of the located object, as a reference object rotated by 90° and a located object placed relatively in front could also be described as “next to” using the intrinsic FOR (see **Figure 1**). Following Graf and Herrmann (1989), we distinguished between vehicle (e.g., chair) and opposite (e.g., shelf) objects that reveal differences in the assignment of the intrinsic left/right axis according to their predominant use. Of the 66 pictures, 36 consisted of vehicle objects (chair, armchair, sofa) in four different orientations (excluding object configurations in which the intrinsic and relative FORs were aligned) and 30 showed opposite objects (wardrobe, bookshelf, chest of drawers). For the opposite objects, only the rotations 0°, 90°, and 270° were used, as these objects are characteristically used with their back to a wall. We distinguished between these object categories in order to control for potential differences in FOR selection.

The randomization procedure took reference objects and their rotation as well as located objects and their positions into account.

### PROCEDURE

Participants were recruited by email invitation, in which they received a link to the online study. First, instructions and three examples were given using objects distinct from those in the study. Afterward, the participants were shown the stimuli and asked to fill in the gaps of the sentences. The whole study comprised 66 trials and lasted about 20 min. Participants could then enter a prize draw for one of 10 prizes of 10 Euros.

### RESULTS^1^

Assuming axis-dependent regularities in FOR selection, we investigated the effect of object rotation and position of the located object on FOR selection. Thus, the descriptions of the participants were coded as using “relative FOR,” “intrinsic FOR,” or “other” (for cases on which no FOR was used). Two rotations (90°, 270°) were used for analysis to ensure a constant dissociation of FORs.

Statistical analyses were performed with R (R Core Team, 2013) using the “lme4” package (Bates et al., 2011). Mixed-effects models of logistic regression for binomially distributed outcomes (generalized linear mixed models, GLMM) were used for the analysis of FOR selection. Mixed-effects models are efficient for the analysis of psycholinguistic data as they allow to include random effects of subjects and items “effectively solving the ‘language as a fixed effect fallacy”’ (Quené and van den Bergh, 2008, p. 413).

**FIGURE 2:**
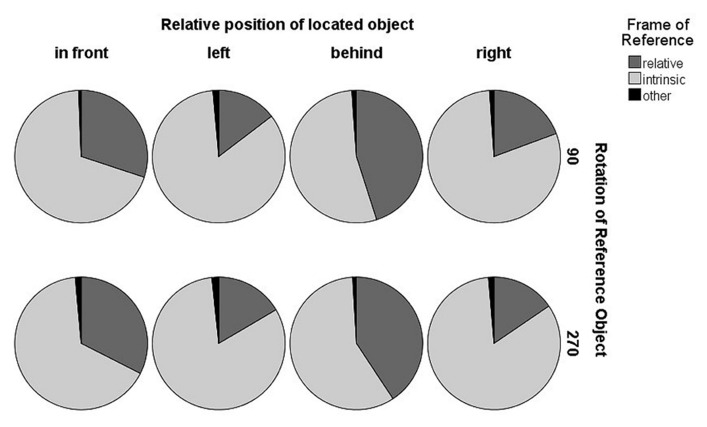
**FOR selection in the first study**.

Descriptions that did not use either FOR were excluded (1.37% of the data). As we only used two rotations of the reference object, the position of the located object was either on the relative or on the intrinsic front/back axis of the reference object. In order to investigate FOR preferences resulting from the position of the located object, we fit a logistic mixed-effects model with position of the located object as fixed effect, full random slopes, and intercepts for subjects and items and FOR selection as dependent variable. Positing the relative front position as intercept, we found significant differences to all other positions (relative left, i.e., intrinsic front/back position: β = 2.61, SE**= 0.3, *z *= 8.66; relative behind: β = -1.37, SE**= 0.19, *z *= -7.05; relative right, i.e., intrinsic front/back position: β = 2.02, SE**= 0.36, *z *= 5.68, all *p < *0.001). These differences in FOR selection resulting from the position of the located object are illustrated in **Figure 2**. Please note that the relative positions “left” and “right” coincide with the intrinsic front–back axis. Regularities of FOR selection suggest an axis-dependent effect, potentially comparable to the distinction between two forms of visuospatial perspective taking (Flavell, 1986). Front/behind judgments are easier to process than left/right relations as they do not require a simulated rotation movement (Kessler and Rutherford, 2010). Furthermore, this axis-effect stands in line with previous research which has shown that the front/back axis is easier to access than the left/right axis due to body asymmetries (Franklin and Tversky, 1990). Additionally, we speculate that the differences between relative “front” and “behind” (i.e., more relative FOR selection when the located object is positioned behind the reference object) might result from the occlusion of the located object. We assume that this occlusion might give more salience to the relative FOR.

We controlled object category in the design in order to eliminate effects of object category on FOR selection. However, when we additionally posited object category as fixed effect in the same model, model comparison revealed no statistically significant effect of object category. This indicates that FOR selection did not differ between vehicle and opposite objects.

### CONCLUSION

Our results reveal a general preference for the intrinsic FOR but also significant effects of the position of the located object. Accordingly, we are now able to differentiate between preferred choice for the object configurations (i.e., the intrinsic FOR) and priming effects.

## STUDY II: PRIMING vs. PREFERENCE

### MATERIALS AND METHODS

#### Participants

Fifty four participants were paid volunteers in the experiment. Due to experimenter error, two groups (four participants) had to be excluded, thus data from 50 participants (11 male, 39 female) ranging in age from 19 to 61 (*M *= 24.3, SD**= 6.2) was used.

#### Stimuli and design

Using a priming paradigm, we constructed prime and target trials in three conditions which only differed with regard to the prime trials. We thus controlled the target trials within items. Altogether, the experiment consisted of 144 prime-target pairs resulting from three priming conditions for each of the 48 target pictures (3 priming conditions × 6 reference objects × 2 rotations × 4 positions of LO).

**FIGURE 3:**
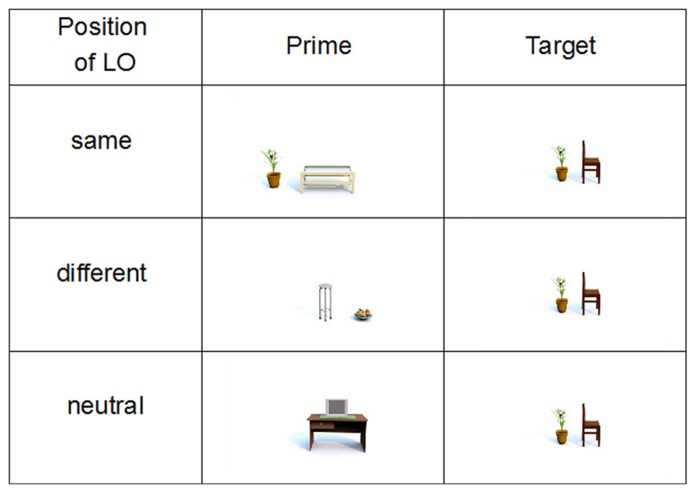
**Example of prime-target pairs in three priming conditions**.

The stimuli were pictures created with indoor planning software (Sweet Home 3D). The pictures showed object configurations, consisting of a reference object and a located object. For the prime trials, three types of pictures were created (33 pictures for each type): *neutral*, *same position*, and *different position*. In the neutral pictures, both FOR were available and aligned as the located object was positioned along the vertical axis of a triaxial reference object. The other two types of pictures (*same* and *different position*) comprised a biaxial reference object and a located object which was positioned on one of the horizontal axes. Accordingly, the intrinsic FOR was unavailable and the participants had to use the relative FOR. In the *same position* condition, the located object was at the same position in prime and target trials (e.g., to the left of the reference object within the relative FOR, see **Figure 3**). In the *different position* condition, the located object was placed at the opposite side of the reference object than in the target trial (e.g., to the right of the reference object in the prime trial and to the left of the reference object in the target trial, both within the relative FOR). The *same* and *different position* conditions were used to test whether priming effects are stronger when the located object has the same position in prime and target trial which would be a plausible consequence of lexical priming, given that the same prepositions would be used.

The pictures described in the first study (in 90° and 270° rotation) were used as target stimuli. See **Figure 3** for an example of the three priming conditions using the same target trial.

Randomization took into account priming condition, the reference object and its rotation, and the position of the located object. To counterbalance the sequential order, the experiment was conducted in two versions by switching the order for half the participants.

**FIGURE 4:**
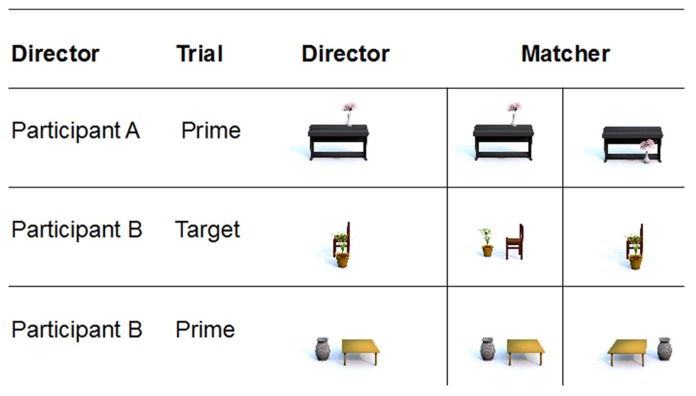
**Role change between participants**.

There were two roles for the participants that changed after every two trials: the director and the matcher. The director was shown a single picture of an object configuration and described it to the matcher while the matcher was shown two pictures and had to decide which of the two fitted the director’s description. The matcher’s two pictures always showed the same reference object at the same rotation as on the director’s screen. However, the position of the located object differed so that the director’s descriptions became potentially ambiguous with regard to FOR in the target trials. Thus, if participant B (director) described the target configuration in **Figure 4** as “The plant is in front of the chair,” either picture could plausibly be correct depending on the matcher’s FOR interpretation. Interpreted within a relative FOR, the picture on the left is correct; interpreted within the intrinsic FOR, the picture on the right is correct. However, as only one of the two pictures corresponded to the director’s picture, its choice revealed whether participants successfully solved the problem of ambiguity.

After every two trials, the roles changed so that the director became the matcher and vice versa. Therefore, the description of the previous director was used as the prime for the description of the subsequent director. Thus, participants took it in turns to prime each other. See **Figure 4** for an example of a prime-target sequence (in the subsequent target trial, participant A would have been the director).

### PROCEDURE

Two naïve participants participated together as interlocutors in a dialog task. Each participant sat in front of a computer screen on which the stimuli were displayed. Participants were separated by a movable wall so that they were able to hear each other but could not see each other nor the other’s computer screen. At the beginning of the experiment, written instructions were presented on the monitor, informing the participants about the procedure of the experiment. Before the start of the study, participants completed five test trials with stimuli distinct from those used in the study. After that, they were asked if the task was clear to them and if so, the study started. The director was shown a single picture whereas the matcher saw two pictures. The director immediately started describing the spatial configuration. The matchers’ task was to determine which of the two pictures matched the director’s description and respond by pressing predefined keys on a button box (left key for left picture, right key for right picture). Accuracy ratings were measured using E-Prime (Psychology Tools Software). The matcher was also allowed to give feedback (e.g., ask the matcher for more information, indicate ambiguities). Both participants’ pictures remained on the screen until a response was given. The whole study comprised 288 trials (144 prime-target pairs) and lasted about 15 min.

The participants were unaware of the objective of the experiment and of the type of trials they were completing. No feedback was given during the experiment.

### RESULTS

Data from 50 participants were used for analysis. Statistical analysis was carried out in PASW Statistics 18 and in “R” (R Core Team, 2013) using the lme4 package (Bates et al., 2011). Mixed-effects models of logistic regression (generalized linear mixed models for binomially distributed outcomes, GLMM) were used for the analysis of FOR selection and accuracy.

Our statistical analysis considered FOR selection in the director’s descriptions of target trial stimuli and the matcher’s accuracy. Furthermore, we conducted a qualitative analysis of the linguistic behavior in terms of the strategies used to disambiguate descriptions. Rationales for each analysis are given in each section.

#### Priming of FOR in dialog

In order to investigate effects of priming or preference on FOR selection, we analyzed the FOR selection in the director’s descriptions. While a prevailing use of the relative FOR in target trials would indicate priming effects, predominant use of the intrinsic FOR would suggest effects resulting from FOR preference.

The director’s descriptions were transcribed and categorized according to FOR use. For categorization, we used the first uninterrupted utterance of the speaker (cf. de Ruiter et al., 2012). In some cases, participants used both FOR at the same time. These descriptions were categorized as “ambiguous” (10.3% of the data). Descriptions that did not use a specific FOR but rather referred to the location of the object on the screen or the proximity of the objects to the director were categorized as “other” (10.6% of the data). Data that revealed participant’s error, for instance when the matchers erroneously described what they saw, were also excluded (0.17%). The rates of FOR selection in prime and target trials are summarized in **Table 1**.

**Table 1:** FOR selection and target trial accuracy.

FOR	Prime trial (%) (*N* = 3600)	Target trial (%) (*N* = 3600)	Target trial accuracy (% within FOR)
Relative	93.8	50	80.8
Intrinsic	0	29	71.8
Ambiguous	0.2	10.3	82.1
Other	4.7	10.6	97.4
NA	1.3	0.2	–

As **Table 2** shows, the relative FOR was chosen more often than the intrinsic FOR. This result was in contrast to the preference for the intrinsic FOR in our first study. Thus, we compared FOR choice between the two studies in order to analyze whether the differences in FOR selection were statistically significant. We fit a logistic mixed-effects model with *study type* (baseline vs. dialog) as a fixed effect and random slopes and intercepts for subjects and items. The results showed a significant main effect of *study type* (β = -3.21, SE**= 0.88, z**= -3.67, *p* < 0.001) confirming the difference in FOR selection between the two studies and revealing more use of the relative FOR in the dialog study.

**Table 2:** Differences in FOR index within and between groups.

	Difference of index scores		
	Mean (SD)	Min	Max
Same group	0.18 (0.33)	0	0.97
Subsequent group	0.37 (0.4)	0	1

In the next step, we investigated whether FOR selection in the dialog study had an effect on target trial accuracy. We assumed that if priming or preference was a prevailing mechanism in order to disambiguate FOR, there should be an effect of FOR selection on target trial accuracy (as a measure for communicative success). Thus, we fit a logistic mixed-effects model with FOR selection as fixed effect and random slopes and intercepts for groups and items. There was no significant effect of FOR choice on target trial accuracy (β = -0.79, SE**= 0.57, z**= -1.39, *p* = 0.17).

#### Differences between priming conditions

As described above, we used three priming conditions (*neutral*, *same position*, *different position*) in order to investigate whether priming effects are stronger when the located object has the same position in prime and target trial. This would be a plausible consequence of lexical priming, given that the same prepositions would be used. Accordingly, we analyzed whether the three priming conditions differed with regard to FOR selection in the target trials. We excluded “other” and “ambiguous” answers and error cases from analysis.

Again, we fit a mixed-effects model of logistic regression positing priming condition as a fixed effect and using random slopes and intercepts for groups and items. Using the *neutral* condition as intercept, there was no significant effect of priming condition (*same* *position*: β = -0.16, SE**= 0.2, *z* = -0.82, *p* = 0.41; *different position *β = -0.39, SE**= 0.23, *z *= -1.74, *p* = 0.08). This reflects that the three conditions did not differ with regard to FOR selection (relative or intrinsic) in the target trials. However, results revealed a marginal difference between the *neutral* condition and the *different position *condition.

#### Effects resulting from the position of the located object

**FIGURE 5:**
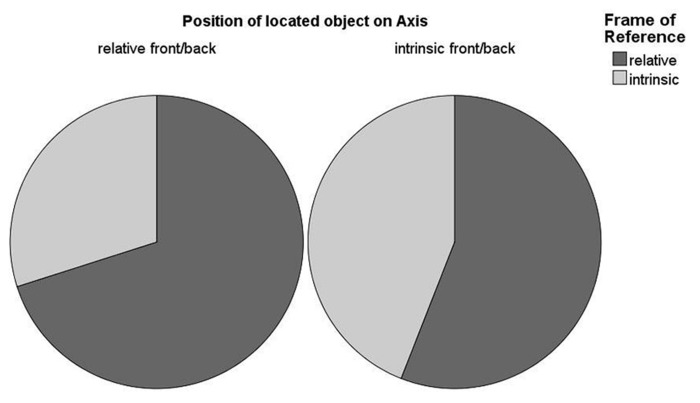
**Effect of the axis position of the located object on FOR selection**.

As the first study had shown that the position of the located object had a significant effect on FOR selection indicating axis-dependent preferences, we analyzed whether this result was replicated in the second study by fitting a mixed-effects model of logistic regression (using only relative and intrinsic FOR descriptions in the target trials). We posited position of the located object as a fixed effect and used full random slopes and intercepts for groups and items. There was a significant main effect of position of the located object*.* Using the relative front position as intercept, there were significant differences compared to position of the intrinsic front and back (i.e., the relative left: β = 1.5, SE**= 0.29, *z *= 5.19 and relative right: β = 1.25, SE**= 0.32, *z *= 3.88, both *p* < 0.001) but not compared to the relative behind position. This indicates that there was a higher amount of relative FOR use when the located object was positioned along the relative front/back axis. Thus, we recoded the positions of the located object in terms of axes so that we were able to distinguish between the relative and intrinsic front/back axes. We then fit a logistic mixed-effects model using *axis* as fixed effect and random slopes and intercepts for groups and items. The results showed a significant effect of the axis (β = 1.26, SE**= 0.28, *z *= 4.51, *p* < 0.001, see **Figure 5**) revealing a greater use of the intrinsic FOR when the located object was positioned on the intrinsic front/back axis.

#### Variability in FOR selection within groups

We expected that participants within groups would adapt the same FOR in order to facilitate mutual comprehension. Thus, we analyzed the variability in FOR selection within and between groups by computing FOR indices (cf. Watson et al., 2006). These indices reflect how similar the descriptions of the participants were with regard to FOR selection within groups, i.e., the more the interlocutors used the same FOR, the lower the FOR index. The FOR indices were computed for each participant by dividing the amount of relative FOR descriptions by the sum of relative and intrinsic descriptions (thus, the analysis excluded the categories ambiguous and other). We subtracted the index from participant B from the index from participant A and squared the result to avoid negative numbers.

To test these within-group indices against indices that would arise between random interlocutors, we subtracted the indices between participants of subsequent groups. These indices from participants that did not engage in a conversation and could not influence each other reflect overall patterns that may arise by chance (see **Table 2**). As the data were not normally distributed (Shapiro–Wilk Test: both df = 25; index within groups S–W = 0.6; index between groups: S–W = 0.78, both *p* < 0.001) we compared the two values using Wilcoxon signed-ranks test and found a significant difference (*Z* = -2.09, *p* < 0.05). The scores between random interlocutors were significantly higher than the scores within groups; this reflects that participants adapted to each other and tended to use the same strategies, independent whatever the specific choice of strategy involved.

**FIGURE 6:**
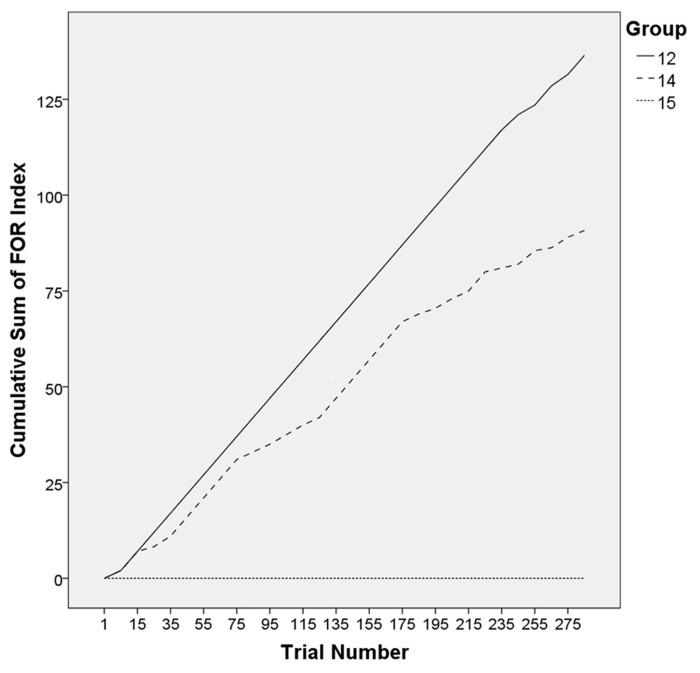
**Exemplary time course of difference scores for three groups**.

**FIGURE 7:**
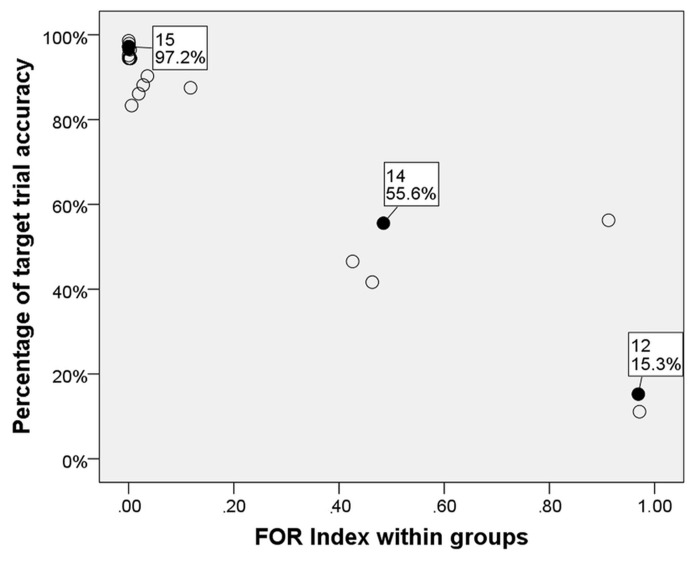
**Scatter plot of target accuracy and difference scores within groups**.

In order to analyze whether the time course of the dialog led to an increased adaptation of strategies between interlocutors, we calculated sliding averages of the FOR indices within groups. Considering a window of four target trials at a time, again, we proceeded as described above for the computation of FOR indices and, again, subtracted the FOR index of participant B from the FOR index from participant A. By shifting the four-trial-window one trial further at a time, we obtained averages that represented how participants adapted their FOR over time. For illustration, we chose three groups as examples (**Figure 6**) revealing different degrees of adaptation (high, medium, and low) between interlocutors (the respective target trial accuracy for the three groups is depicted in **Figure 7**).

Next, we assumed that a mutual adaptation of FOR reduced misunderstandings thus leading to a more efficient communication (measured here as target trial accuracy). We analyzed whether sliding average FOR indices within groups had an effect on target accuracy. We fit a mixed-effects model of logistic regression with target trial accuracy as dependent measure, FOR indices as fixed effects and full random slopes and intercepts for groups and items. We found a significant main effect of index scores (β = -1.38, SE**= 0.3, *z *= -4.68, *p* < 0.001). For an illustration of the relationship between overall target trial accuracy and FOR index scores within groups, see **Figure 7** (case labels include group number and overall target accuracy for the three exemplary groups depicted in **Figure 6**).

### QUALITATIVE ANALYSIS

In a qualitative analysis of the data, we investigated which qualitative strategies interlocutors applied to resolve ambiguities in target trials, considering only trials in which the relative or the intrinsic FOR were used. We analyzed the annotated dialog considering more than the first uninterrupted utterance for additional strategies to solve FOR ambiguities. Our analysis showed that additional information for disambiguation was provided in 23.3% of the target trials by the director even though the matcher indicated that the description was ambiguous with regard to the spatial FOR in only 1.5% of the target trials.

However, 11 out of 25 groups did not provide any additional information. Their results varied with regard to the matcher’s accuracy (mean 78.8%, SD 30.6, ranging from 11 to 99%) and difference scores (mean 1841.7, SD 2690.5, ranging from 0 to 7448). The other 14 groups varied in their strategies, although we could classify three main approaches. The most common was a definition of the perspective (in 13.8%, e.g., “In front of the chair, as seen from my/the chair’s perspective”). Other strategies were reference to specific intrinsic features of the object (8.3%, e.g., “The stool is in front of the front of the couch”) or the use of specific verbs (1.3%) to express the position of the located object (e.g., “The plant disappears behind the sofa”). The latter was, however, only used in trials in which the located object was positioned relatively behind the reference object and was thus partly covered by it.

With regard to the quantity of strategies within each FOR, we found that more additional strategies were used within the intrinsic FOR (39.7%) than within the relative FOR (13.4%). Within the intrinsic FOR, 25.2% of the target trial descriptions contained additional information with regard to the perspective (7.3% within the relative FOR).

### CONCLUSION

Our results reveal a general priming effect of the relative FOR (as shown by the comparison between the two studies) and a significant effect of the located object’s position on FOR selection. There were, however, no significant differences between the three priming conditions.

Furthermore, our results show that participants adapt each other’s strategies (as shown by the comparison between intra- vs. intergroup difference scores) and that target trial accuracy is influenced by the extent of this adaptation.

With regard to qualitative strategies, we found that even though FOR ambiguity was indicated in only 1.5% of the target trials, participants added further information in about a third of the target trials (27.8%). Strategies comprised perspective marking (17.2%), the reference to intrinsic features of the reference object (9.4%) or the use of verbs denoting a specific location (1.2%). Strategies were used more often within the intrinsic FOR (39.9%) than within the relative FOR (13.6%).

## DISCUSSION

In the present study, we investigated effects of priming and preference on FOR selection in a dialog task. As the prime trials only allowed a description using the relative FOR (i.e., the intrinsic FOR was not available or both FOR were aligned), the priming account would predict a prevailing use of the relative FOR in the target trials (cf. Pickering and Garrod, 2004; Watson et al., 2004), even though the intrinsic FOR was available. The comparison of FOR selection (intrinsic vs. relative) between the first and the second study revealed significant differences indicating greater use of the relative FOR in the dialog study. This increase in the use of the relative FOR might reflect priming effects in target trials resulting from processing the relative FOR in the preceding prime trial. In any case, the preference for the intrinsic FOR, as found in the first study, was diminished, which indicates that this preferences cannot be considered robust and predominant (contra to Miller and Johnson-Laird, 1976). Interestingly, the choice of FOR had no effect on target trial accuracy. In order to efficiently solve FOR ambiguities, we would have expected that participants negotiated which perspective should be used, comparable to a conceptual pact (Brennan and Clark, 1996) with regard to the spatial FOR. However, no group used this strategy and defined a consistent perspective. This indicates that the groups must have used other strategies.

Even though priming effects might explain the more frequent choice of the relative FOR, we would like to discuss the role of priming in communication. Priming leading to alignment has been claimed to be the key to successful communication (cf. to Pickering and Garrod, 2004). In our study, the primed relative FOR was used in target trial descriptions only half of the time. If priming was automatic and thus unavoidable, should we not expect a greater frequency of relative FOR selection in target trials? Given that half of the time, interlocutors did *not* use the primed FOR, the role of priming as the prevailing mechanism in communication might have been overestimated. Furthermore, FOR selection did not have an influence on target trial accuracy. If both interlocutors were primed to use the same FOR, this should be evident not only in their spatial descriptions but also in their interpretations of the other’s descriptions. Thus, our findings indicate that, even though priming may have an influence on FOR selection in dialog, it may not be as automatic and comprehensive as has previously been assumed and does not necessarily lead to successful communication (measured here in terms of target trial accuracy; cf. Pickering and Garrod, 2004).

Despite the fact that there was a general priming effect, there were no differences between the three priming conditions (*neutral* vs. *same position* vs. *different position* of the located object) with regard to the FOR selection in the target trials. This suggests two things: on the one hand, it did not matter whether the relative position of the located object was the same in prime and target trial. Thus, accessing single components of the relative FOR (i.e., the front/back axis) leads to an activation of the whole FOR resulting in a priming effect in the subsequent trial. As the intrinsic FOR was either not available or aligned with the relative FOR in the prime trials, we can exclude an inhibition of the FOR as reported by Carlson and Van Deman (2008). However, due to the design of the experiment, our focus was on activation of FOR, which limits our conclusions about the nature of inhibition. On the other hand, the fact that there was no difference in effects of FOR selection between the *same* vs. *different position* condition reveals that there was no cumulative effect of lexical and FOR priming, a result that supports findings previously reported (Watson et al., 2004).

Independent of priming effects, we found effects on FOR selection resulting from the position of the located object in both studies. If the located object was positioned on the front/back axis of the FOR (relative or intrinsic), this made the choice of the respective FOR more likely. This suggests a general preference for localizing along the front–back axis and stands in line with related work. With regard to the egocentric FOR, this result coheres with the idea that the front/back relations are easier to process, due to the inherent asymmetric features, than are left/right relations, as has been reported before (e.g., Tversky, 1996). With regard to perspective taking, fundamental differences in processing the front/back compared to the left/right axis have been reported (e.g., Kessler and Rutherford, 2010). Extending these findings with regard to the intrinsic FOR, our results emphasize the impact of the intrinsic front/back axis in spatial descriptions.

As we have shown that priming effects were less pronounced than we would have expected from an automatic process and that FOR selection did not have an effect on target trial accuracy, we assumed that the groups developed their own strategies to resolve FOR ambiguities. In order to investigate these strategies, we calculated difference scores within groups that represented how similar the descriptions of the two interlocutors were and compared them to difference scores that arose between random interlocutors. The significant difference between the groups revealed that within groups, interlocutors tended to adopt the same strategies, using either the relative or intrinsic FOR, both FOR at the same time or other descriptions which completely avoid spatial FOR. This indicates that interlocutors adapted to each other, but not necessarily by consistently using the primed relative FOR or the preferred intrinsic FOR. The efficiency of this mutual adaptation of strategies was measurable in terms of target trial accuracy: the more interlocutors adapted each other’s strategies, the more accurate they were. More generally speaking, this reveals that communicative success depends on mutual adaptation. A comparable adaptation process of types of descriptions has been reported for players in a maze-game (Garrod and Anderson, 1987). Furthermore, Schober (1993) found that pairs of interlocutors in dialog varied idiosyncratically with regard to the perspective-setting strategies they used in their descriptions of spatial configurations. Under these conditions, a lot of variability between groups was possible without impairing the ultimate success of communication. This variability is necessarily reduced in dialog studies in which one of the interlocutors is a confederate. While the naïve participant can adapt to the confederate’s strategies, the confederate’s contributions are limited to scripted utterances. Thus, the collaborative aspect of communication that arises from the fact that “language use is really a form of joint action” (Clark, 1996, p. 3) becomes a unilateral process. This reduction in variability may explain why priming effects appear stronger in such studies.

Interestingly, there were five groups in our study that revealed a very low level of adaptation (i.e., very high difference scores) and a target trial accuracy equal to or below 56%. The low percentage of accuracy reveals that interlocutors misunderstood each other about half of the time (or even more often for lower numbers). By taking a closer look at the strategies of each participant, we found that all five groups showed the same pattern: one of the participants predominantly used the relative FOR whereas the other participant used the intrinsic FOR. This pattern may reflect individual preferences, as pointed out by Levelt (1982). Given that the experiment did not include feedback with regard to accuracy and that both target pictures could possibly be correct within different FOR interpretations, participants obviously did not realize that they used different FOR throughout the dialog. We avoided including feedback in order to allow participants to develop their own strategies for dealing with the problem of FOR ambiguity and to keep the dialog as natural as possible. Thus, misunderstandings resulting from different FOR interpretation may be common in natural language (20% of the groups experienced this problem).

Following this idea, we investigated the time course of dialog with regard to difference scores. When plotting the cumulative sum of these scores over the trial sequence, the slope of the resulting curve depends on the difference score: the higher the score, the steeper the curve. In general, groups that revealed a high level of adaptation showed a low slope of the resulting curve whereas groups that adapted each other’s strategies to a lesser extent revealed a steeper curve. Taking three groups as examples that differed with regard to their target trial accuracy, we found that the more successful the group was (i.e., in maintaining overall high target trial accuracy), the lower the difference scores remained over time, indicating a constantly high level of strategy adaptation (see, group 10). As expected, the opposite pattern was found in unsuccessful groups (i.e., revealing overall low target accuracy) that showed high difference scores throughout the dialog, reflecting that participants consistently used different strategies. Group 12 (**Figure 6**), for example, showed this opposite pattern: constantly high difference scores arose due to different descriptions strategies between interlocutors, leading to a steep increase of the curve over time and low target trial accuracy (15.3%). Group 14 can be considered as being moderately successful with a target accuracy of 55.6%. Note that this percentage indicates that participants misunderstood each other nearly half of the time.

However, even though we can conclude that mutual adaptation of strategies seems to be strongly facilitating communicative success, an open question remains why some groups showed high levels of adaptation while other groups did not adapt at all. This question cannot be answered unambiguously but there may be two explanations. Haywood et al. (2005) have shown that speakers in a dialog study were sensitive to the ease of comprehension for their interlocutor, disambiguating their descriptions in visually ambiguous contexts. Thus, on the one hand, the lack of disambiguation in some of the groups in our study could reveal that participants erroneously assumed they were successful because they failed to notice the potential ambiguity. This stands in line with the claim that people are normally not aware of the fact that there are two alternative FOR (Grabowski and Miller, 2000, p. 526). On the other hand, we cannot exclude that participants may have deliberately chosen not to adapt to each other’s strategies, for instance due to a lack of motivation for solving the task successfully. Thus, collaboration may well be a prerequisite for successful communication.

In a final step, we investigated the dialogs for qualitative strategies. Qualitative strategies consisted of explicitly adding a perspective to the FOR (e.g., “[...] as seen from my/the chair’s perspective” or “[...] if you sit in the chair”), reference to intrinsic features of an object (e.g., “The plant is behind the backrest of the chair”), or the use of specific verbs (e.g., “The plant disappears behind the chair”). Qualitative strategies were used in nearly one third of the descriptions in addition to the intrinsic or relative FOR. The use of these strategies suggests that the director was aware of the ambiguity problem and tried to help the matcher by adding unambiguous information to resolve it. The fact that this was done quite often may result from the role switching in the dialog. As both interlocutors were confronted with FOR ambiguities when they were the matcher, they were aware of the problem when they were director.

Interestingly, about a quarter of the descriptions within the intrinsic FOR contained information about the perspective and thus the Origo of the FOR. This stands in contrast to what has been assumed by Grabowski and Miller (2000, p. 521) who claimed that “the entity that constitutes the Origo is never expressed explicitly in the case of intrinsic relations.” By contrast, the infrequent addition of explicit perspective in trials in which the relative FOR was used is surprisingly small given the prediction that “[...] if a deictic^2^ interpretation is intended when an intrinsic interpretation is possible, the speaker will usually add explicitly ’from my point of view’ [...]” (Miller and Johnson-Laird, 1976, p. 398). We assume that giving information on the Origo depends on the speaker’s confidence about the listener’s interpretation of the FOR and can be interpreted in terms of the Gricean maxims of conversation (Grice, 1975). If both interlocutors have adopted the same FOR consistently, mentioning the Origo would violate the Gricean maxim of quantity and make the contribution more informative than required (Grice, 1975, p. 308). However, when there is no such agreement on a specific FOR, providing no information on the Origo disregards the maxim of manner, i.e., avoiding ambiguity. Thus, we suggest that adding perspective reflects the speaker’s degree of certainty of the listener’s FOR interpretation independent of the type of FOR being used.

In conclusion, our results show that neither FOR preferences nor priming alone represent the key to successful communication in this domain. Intrinsic FOR preferences (as shown in the first study) were partly diminished by priming effects in the dialog study. However, priming effects could only account for half of the FOR selection in target trials. As groups varied widely with regard to their description strategies, priming of FOR leading to an alignment of situation models (Pickering and Garrod, 2004; Watson et al., 2004) does not provide a comprehensive account of successful communication. Rather, successful communication seems to depend on the adaptation of strategies between interlocutors: the more the interlocutors adapted to each other’s strategies, the more successful they were.

## Conflict of Interest Statement

The authors declare that the research was conducted in the absence of any commercial or financial relationships that could be construed as a potential conflict of interest.

## Footnotes

1 Parts of these results have been published (Ziegler, Johannsen, Swadzba, de Ruiter, and Wachsmuth, 2012)

2 In this case, the deictic FOR coincides with the relative FOR in our terminology
